# Supplementary material for: Anipose: A toolkit for robust markerless 3D pose estimation
Source: Cell Rep. Author manuscript; Available in PMC 2021 Oct 8. (PMC8498918; doi:10.1016/j.celrep.2021.109730)
Supplement: 1 [file NIHMS1744538-supplement-1.pdf]

Cell Reports, Volume *36*

Supplemental information

Anipose: A toolkit for robust markerless 3D pose estimation

Pierre Karashchuk, Katie L. Rupp, Evyn S. Dickinson, Sarah Walling-Bell, Elischa Sanders, Eiman Azim, Bingni W. Brunton, and John C. Tuthill

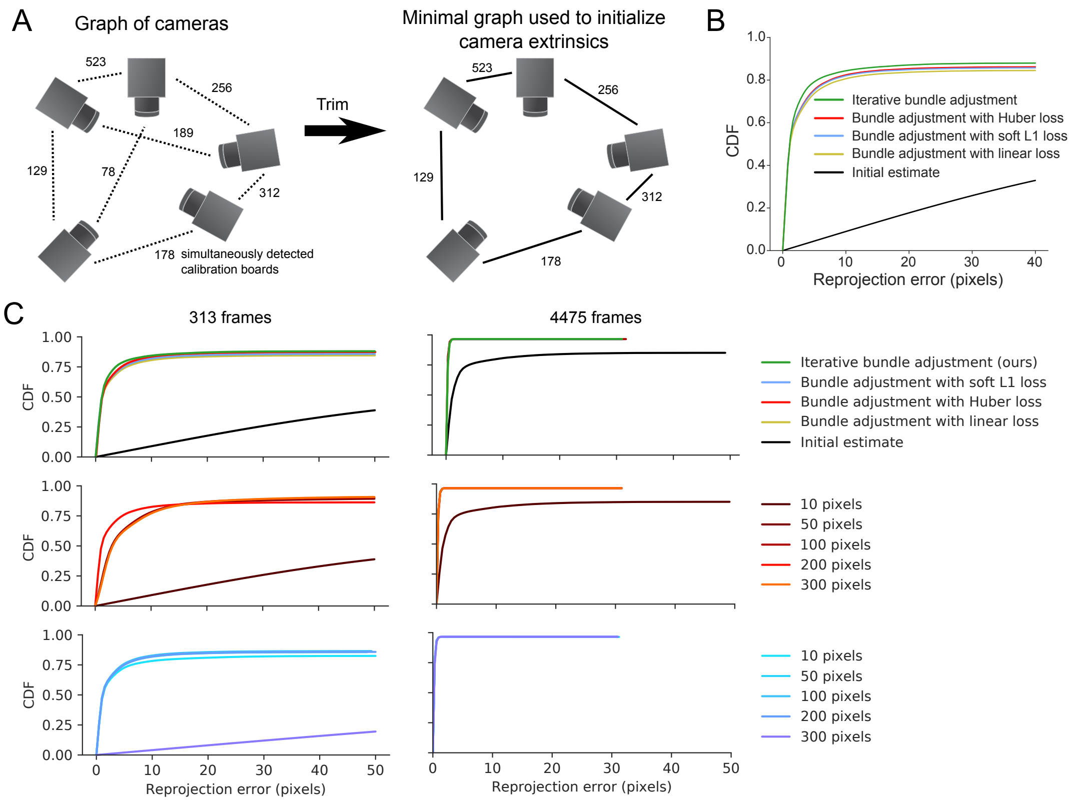

Figure S1: Related to Figure 2B. (A) Illustration of the camera parameter initialization procedure. We build a graph with each camera as a node and edge weights computed by the number of frames the calibration board is simultaneously detected by pairs of cameras. To initialize the camera calibration, we trim this graph to be a minimal, fully connected tree using a greedy approach. (B) On calibration videos from the fly dataset, bundle adjustment improves the initial calibration estimate, as measured by a reduction in reprojection error. (C) Reprojection error as a function of outlier threshold for bundle adjustment with Huber and soft L1 losses.

	ChArUco	Mouse	Fly	Human
Training frames	1200	2200	6632	636724
Test frames	1200	400	1200	159181
Num cameras	6	2	6	4
Pixel scale (mm)	0.0075	0.0897	0.0075	4.79
**2D filter**				
score threshold			0.05	0.05
n_back			3	3
medfilt			13	13
offset_threshold			15	30
spline			true	true
**3D filter**				
score_threshold	0.3	0.3	0.3	0.3
reproj_error_threshold			5	5
scale_length			3	1.5
scale_length_weak			0.5	0.5
scale_smooth			2	4
n_deriv_smooth			3	2

Table S1: Anipose configuration parameters used in this paper. Related to Figures 4 and 5.

number of cameras	filter type	mean joint angle error (deg)	mean joint position error (mm)
2	No filters	11.5	84.3
	Viterbi filter	10.6	76.9
	Spatiotemporal reg. + Viterbi filter	12.0	98.8
3	No filters	7.5	42.6
	Viterbi filter	7.3	41.7
	Spatiotemporal reg. + Viterbi filter	7.1	40.2
4	No filters	6.9	37.0
	Viterbi filter	6.7	36.8
	Spatiotemporal reg. + Viterbi filter	6.4	33.1

Table S2: Estimates of error with different number of cameras for the human dataset. Related to Figure 5.

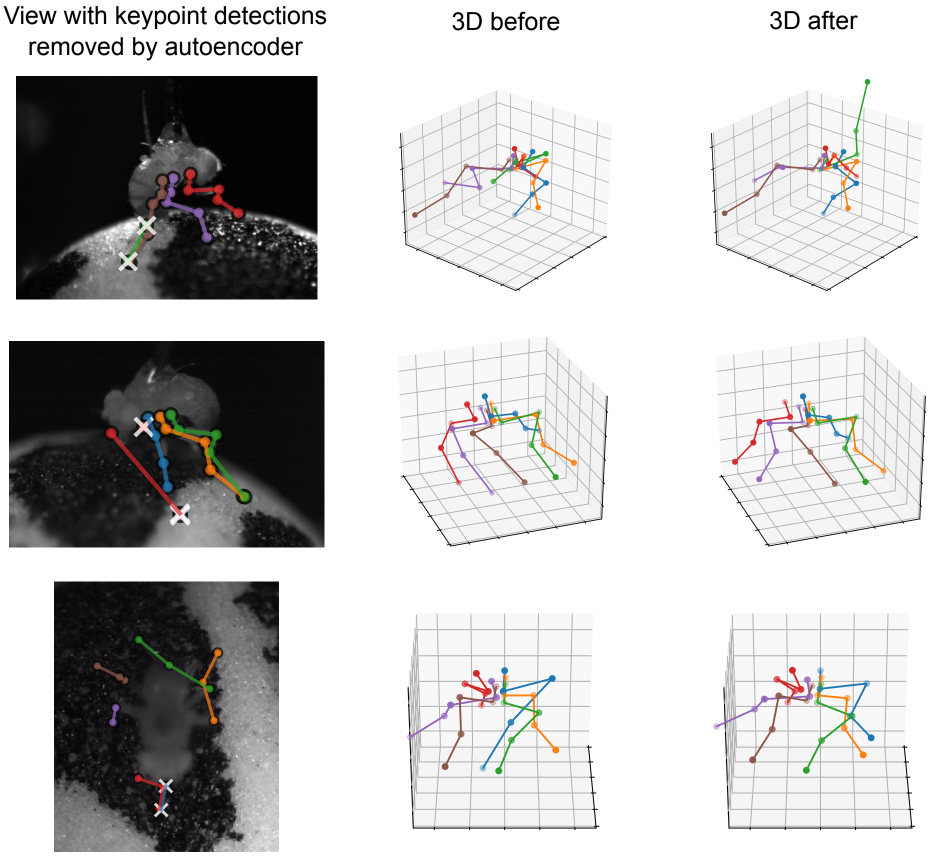

Figure S2: An autoencoder corrects 3D tracking by removing bad keypoint detections. Related to Figure 4. On the left is one view where the autoencoder lowered the confidences for particularly bad detections, thus removing them from the 3D triangulation. On the right are the 3D positions of the keypoints before and after the removal.

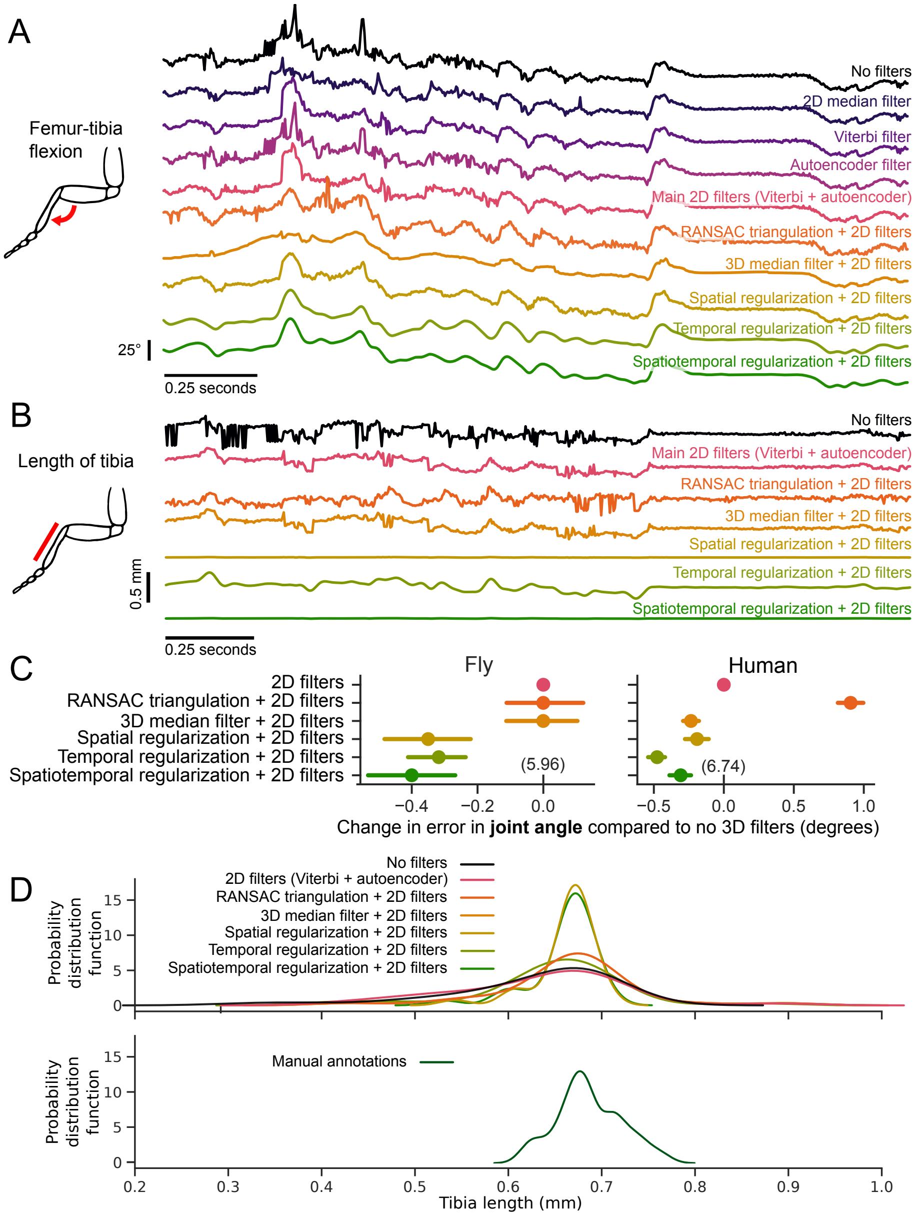

Figure S3: Related to Figure 5. (A) Example traces of the tracked hind-leg femur tibia flexion angle, before and after filtering. (B) Estimation of tibia length over time, before and after filtering. Adding spatial regularization leads to a more stable estimate of the tibia length across frames. (C) Comparison of angle estimates before and after filtering. The mean difference and confidence intervals are plotted as in C. Spatial and temporal regularization improve angle estimation above 2D filters on both datasets ($p < 0.001$, paired t-test). The 3D median filter improves angle estimation on the human dataset ($p < 0.001$, paired t-test) but not on the fly dataset ($p > 0.8$, paired t-test). RANSAC triangulation does not improve angle estimation for either dataset. (D) Comparison of methods for estimating tibia length. Spatial regularization most closely matches the distribution of tibia lengths based on manual annotations. The plots show the distribution of tibia lengths for one fly, extending the example shown in Figure 5B, for different filtering strategies (top) and manual annotations (bottom).

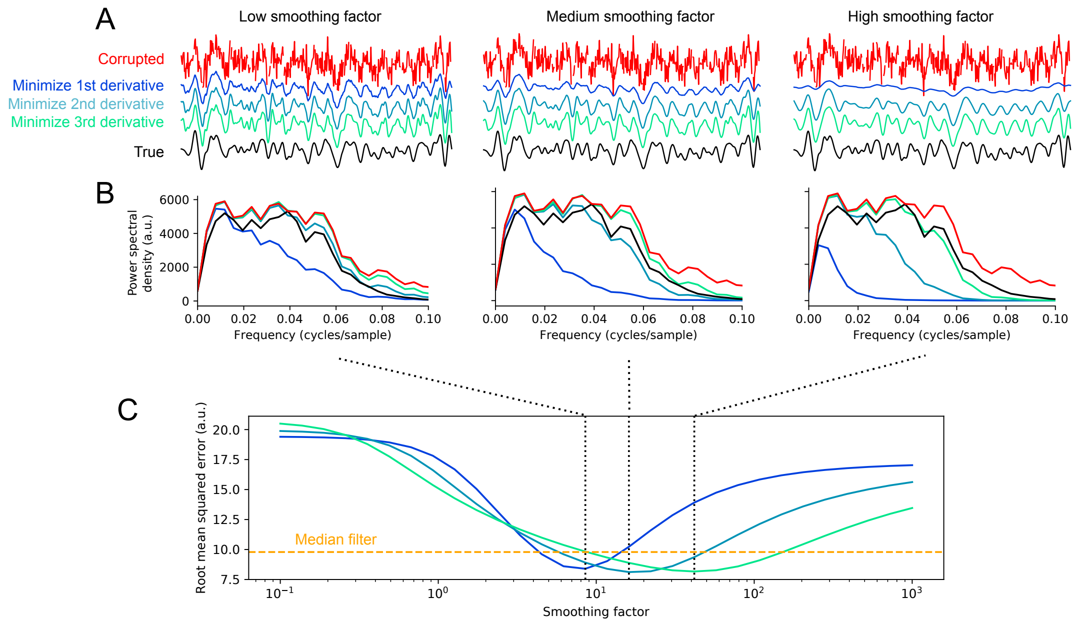

Figure S4: Minimizing higher order derivatives preserves high frequency dynamics and leads to lower reconstruction error. Related to Figure 5. (A) An example simulated trajectory along with its reconstructions using temporal regularization with different derivatives minimized. Each column shows reconstructions with different smoothing factors. (B) We synthesized 30 different trajectories with the procedure in A and compared the average power spectral density between the true, corrupted, and reconstructed trajectories with different derivatives minimized. At any smoothing factor, minimizing higher derivatives preserves more power at high frequencies. (C) The average root-mean squared error (RMSE) of reconstruction for the 30 simulated trajectories. The minimum error for a median filter (over all possible filter widths) is shown as a dashed line, for reference. Dotted lines indicate the smoothing factors shown in A and B. Note that minimizing higher derivatives is more robust to smoothing factor choice, as a wider range of factors give lower RMSE than a median filter. The best RMSE over all possible smoothing factors is lower when minimizing the 3rd derivative than 2nd or 1st.

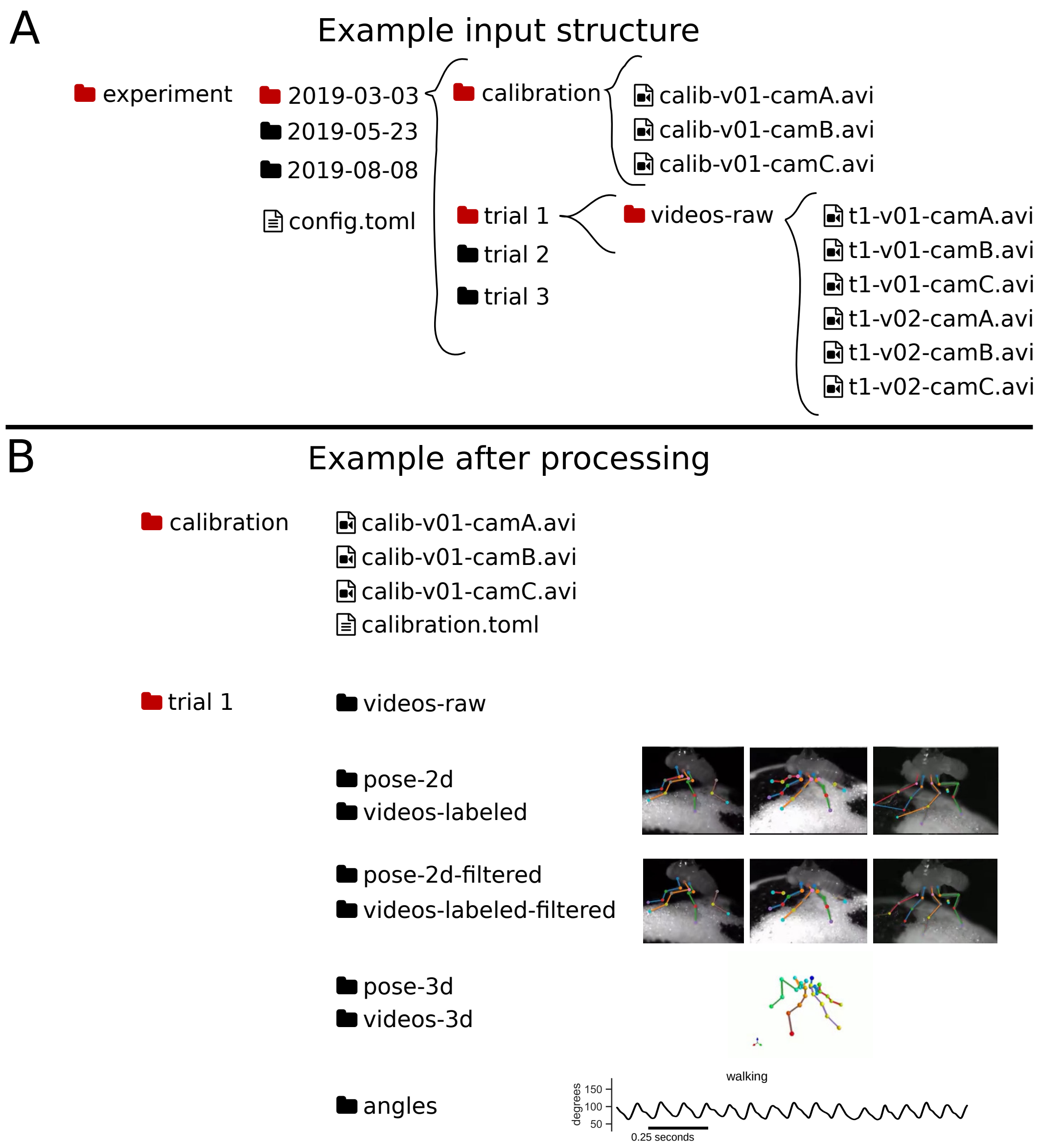

Figure S5: An example of the Anipose file structure. This structure enables visualization of arbitrary datasets, as shown in Figure 6. (A) The input file structure consists of folders nested to arbitrary depths (e.g. "experiment/2019-03-03/trial 1") with a folder for raw videos at each leaf of the directory tree. The calibration folder may be placed anywhere and will apply recursively to all folders adjacent to it. (B) When the user runs Anipose, it will create a folder for each step of processing. New folders created include "pose-2d" and "videos-labeled" which contain the unfiltered keypoint detections and visualizations of those, "pose-2d-filtered" and "videos-labeled-filtered" which contain the filtered keypoint detections and visualizations, "pose-3d" and "videos-3d" which contain the triangulated 3D keypoint detections and visualizations of these, and finally "angles" which contains angles computed based on the 3D keypoint detections.

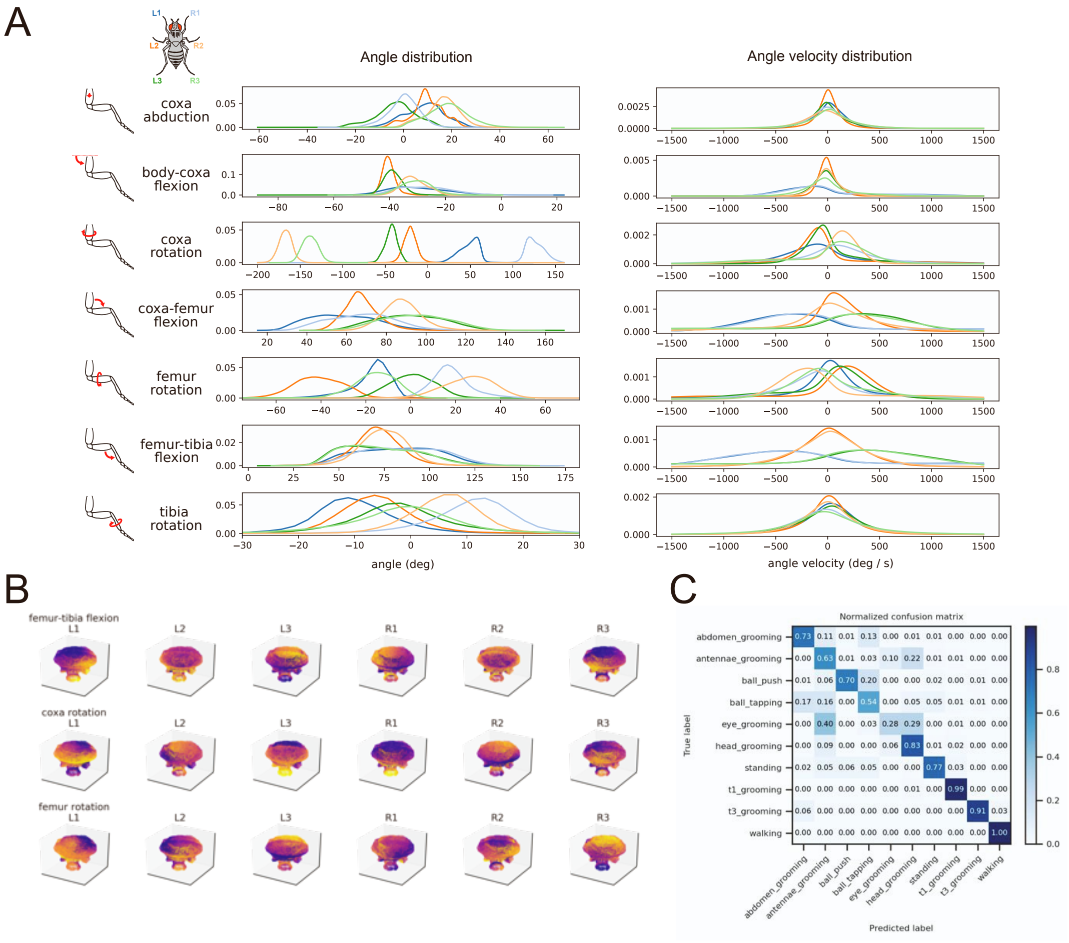

Figure S6: Related to Figure 7. (A) Probability density functions of all joint angles and derivatives for 39 wild type flies during walking, extending the subset of angles presented in Figure 7B. (B) UMAP embeddding of fly walking, as in Figure 7C, colored by each of the joint angles. The colormap is normalized to the angle within each plot. (C) Confusion matrix for the behavior classifier used to isolate walking bouts for Figure 7.

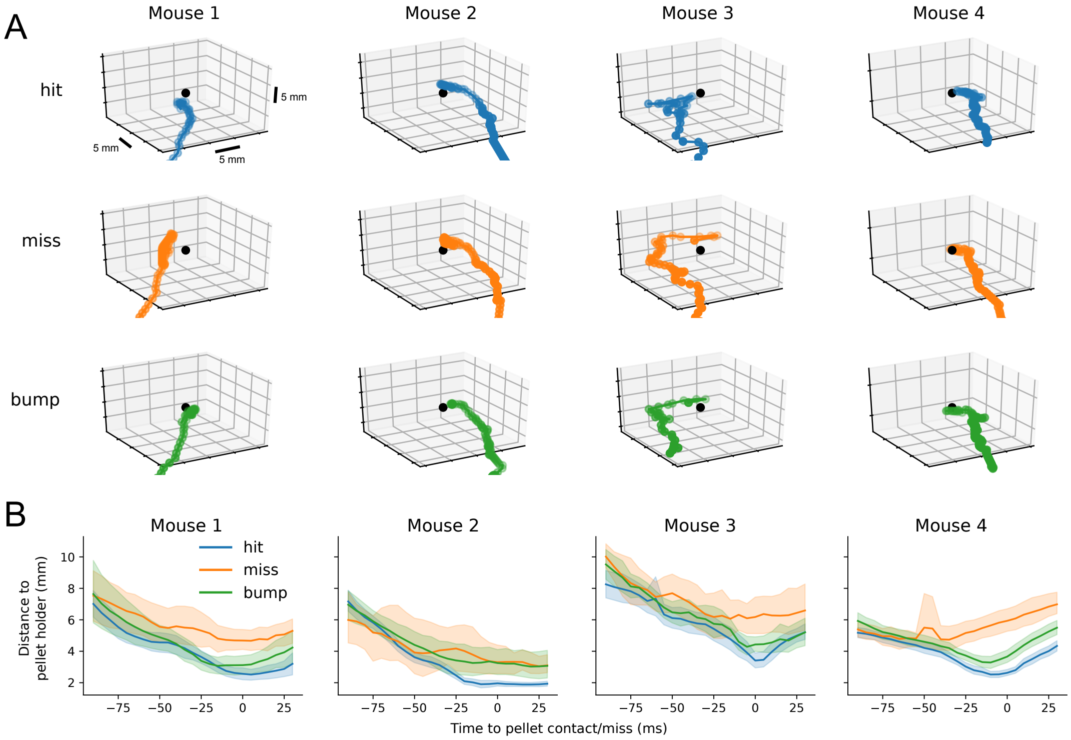

Figure S7: Related to Figure 7. 3D tracking with Anipose reveals common structure of mouse reaches. (A) 3D trajectories of example reaches of each type. The pellet holder is indicated as a black dot. (B) Mean distance to pellet holder as a function of time, for each mouse. Shaded areas are 95% confidence intervals. When reaches are aligned to grasp attempt (0 ms), the hand is farther from the pellet on miss trials compared to hit or bump trials.

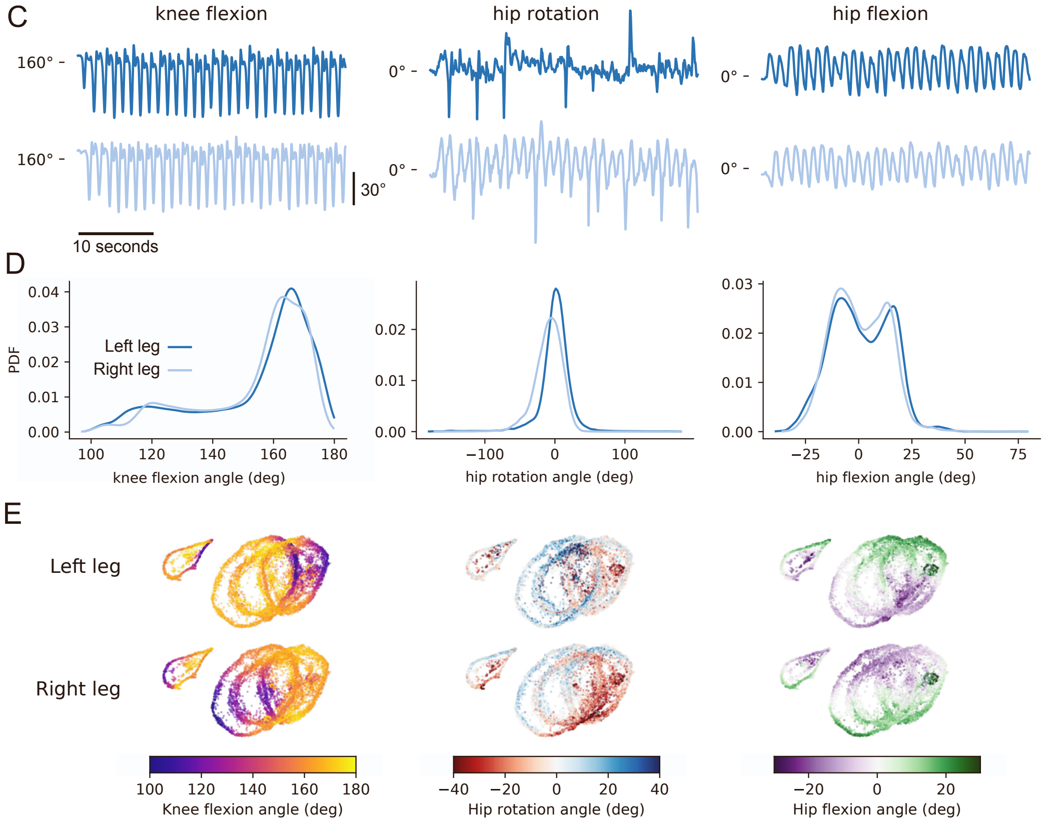

Figure S7: (Continued from previous page). 3D tracking of human walking enables quantification of leg angles and comparison across individuals. (C) Representative traces of knee flexion, hip rotation, and hip flexion from a walking human, tracked with Anipose. Data is from the Human 3.6M dataset. The median angle value is indicated at left as a reference point. (D) Probability distribution functions of knee flexion, hip rotation, and hip flexion angles from 7 humans. Only sessions that include walking are included. Note the asymmetry in the distributions of knee flexion and hip flexion, revealing the known non-sinusoidal pattern of knee and hip flexion during walking. (E) UMAP embedding of knee flexion, hip rotation, and hip flexion angles across all legs, and their derivatives. The UMAP embedding is colored by knee flexion and hip rotation for each leg. Coloring by knee flexion angle reveals the common phase alignment of the circles across subjects. From this phase alignment, we see that the trajectory of hip rotation for each subject is markedly different.
